# Nutrient Supply Gradients Modulate Cultivation-Driven Restructuring of Microbial Communities in Desert Soils

**DOI:** 10.3390/biology15100755

**Published:** 2026-05-09

**Authors:** Feng Wen, Xiru Chen, Qiannan Chen, Lianqiang Li, Yingying Zhao, Yongxia You, Shihao Niu, Zhanfeng Xia

**Affiliations:** State Key Laboratory Incubation Base for Conservation and Utilization of Bio-Resource in Tarim Basin, College of Life Sciences and Technology, Xinjiang Production & Construction Corps, Tarim University, Alar 843300, China

**Keywords:** nutrient supply gradient, oligotrophic cultivation, microbial community composition, Taklimakan Desert

## Abstract

Understanding how cultivation conditions influence the recovery of microbial diversity is critical for interpreting cultivation-derived communities in relation to in situ microbial ecology, particularly in nutrient-limited environments such as desert soils. In this study, we investigated how an oligotrophic nutrient gradient shapes cultivation outcomes and enrichment-derived bacterial community structure in soils from the Taklimakan Desert. Three enrichment treatments representing decreasing nutrient availability—standard low-nutrient medium (R2A), diluted medium (DR2A), and sterile water—were used to compare enrichment-derived communities with the original soil microbiome. Amplicon sequencing revealed that cultivation substantially altered community composition and reduced both taxonomic richness and diversity relative to the original soil. Across enrichment treatments, bacterial communities were dominated by a limited number of taxa, whereas the original soil contained a broader range of low-abundance lineages. Within the enrichment system, variation in nutrient supply influenced the relative abundance of specific taxa, with differential responses observed at the genus level. In contrast, beta diversity analysis showed only limited separation among enrichment treatments, and co-occurrence network analysis indicated generally simple and weakly connected community structures across all conditions. Overall, our results demonstrate that cultivation represents the primary selective force shaping enrichment-derived bacterial communities, while nutrient supply intensity acts as a secondary modulator of taxon-specific enrichment. These findings highlight the importance of incorporating nutrient gradients into cultivation strategies to improve ecological interpretation and facilitate the recovery of oligotrophy-associated microorganisms from desert soils.

## Simple Summary

Desert soils are characterized by chronic nutrient limitation, and many microorganisms in these environments must persist under oligotrophic conditions. In this study, we examined how an oligotrophic nutrient gradient influences cultivation outcomes for desert soil bacteria from the Taklimakan Desert. Using three enrichment treatments representing decreasing nutrient availability—R2A, diluted R2A (DR2A), and sterile water—we compared enrichment-derived communities with the original soil communities. Our results showed that cultivation strongly reshaped bacterial community composition and reduced overall diversity relative to the original soil. With specific genera exhibiting treatment-dependent enrichment patterns, for example, Aquincola showing higher relative abundance under lower nutrient conditions (oligo and water), whereas Acinetobacter was more abundant in the R2A treatment. In contrast, differences among enrichment treatments were less pronounced at the whole-community level, and network topology remained generally simple across treatments. Overall, these findings indicate that cultivation is the primary factor shaping enrichment-derived bacterial communities, whereas nutrient supply acts as a secondary but ecologically meaningful modulator of enrichment outcomes. Incorporating nutrient gradients into cultivation design may improve the ecological interpretation of enrichment results and help refine strategies for recovering oligotrophy-associated microorganisms from desert soils.

## 1. Introduction

Arid desert soils are characterized by persistent nutrient limitation, low organic carbon inputs, and limited water availability, which impose strong constraints on microbial growth and community assembly [1,2,3]. Under such conditions, microbial communities are structured primarily by resource scarcity and environmental stress, leading to the predominance of taxa adapted to oligotrophic and stress-tolerant lifestyles [4,5]. Large-scale culture-independent surveys have described the taxonomic composition and diversity patterns of desert soil microbiomes across spatial gradients [6,7], providing valuable insight into microbial distribution under extreme aridity. However, although in situ community composition has been extensively characterized, considerably less attention has been given to how nutrient availability influences cultivation-based enrichment of these communities.

Microbial cultivation remains essential for isolating functional strains and experimentally linking microbial identity to ecological traits. Conventional cultivation strategies frequently rely on relatively nutrient-rich media, which can preferentially select fast-growing or copiotrophic organisms [8,9]. Such conditions may not accurately reflect the resource-limited environment of desert soils and may bias recovery toward taxa that are competitive under high substrate concentrations. To improve the isolation of slow-growing or oligotrophic microorganisms, diluted and low-nutrient media have increasingly been applied [10,11]. These strategies are consistent with the oligotroph–copiotroph framework, which links resource availability to microbial life-history strategies and substrate affinity kinetics [12,13]. Nevertheless, most studies implement low-nutrient cultivation as a single treatment rather than systematically varying nutrient concentration. Consequently, nutrient availability is rarely treated as an explicit experimental gradient during enrichment, and its quantitative influence on cultivation-driven community restructuring remains insufficiently evaluated [10,11,12,13,14].

From a community assembly perspective, nutrient availability functions as an important environmental filter in desert soils [3,15]. Ecological theory predicts that stronger resource limitation constrains the number of taxa capable of growth under given conditions and may influence interaction structure and co-occurrence patterns [16,17]. Under cultivation, variation in nutrient supply is therefore expected to differentially select subsets of the original soil community. Lower nutrient levels may favor taxa characterized by high substrate affinity and slower growth rates, whereas higher nutrient availability may promote copiotrophic organisms with rapid growth kinetics. Despite these expectations, empirical assessments of graded nutrient supply as a driver of enrichment-derived community restructuring remain limited, particularly in hyper-arid systems such as the Taklimakan Desert [6,7].

The Taklimakan Desert represents one of the most extreme arid environments, characterized by minimal precipitation, strong evaporation, and low soil organic matter content [1,18]. Microbial communities in this region experience chronic resource limitation and strong abiotic filtering. Evaluating how nutrient supply level influences enrichment-derived community composition in such soils is therefore important for interpreting cultivation bias and for improving recovery of ecologically representative taxa.

Despite increasing interest in oligotrophic cultivation, nutrient availability is typically treated as a binary or categorical variable rather than a continuous ecological gradient. As a result, the extent to which different levels of nutrient limitation quantitatively shape cultivation-driven community restructuring remains unclear. In this study, we address this gap by explicitly framing nutrient supply as a graded ecological filter and evaluating how variation in nutrient intensity influences multiple dimensions of enrichment-derived microbial communities. We further examine whether different aspects of community structure (taxonomic composition, diversity metrics, and network topology) exhibit consistent or decoupled responses to nutrient gradients. We hypothesize that nutrient supply acts as a secondary but structured filter during cultivation, selectively reshaping taxon-level composition and interaction patterns without necessarily inducing large shifts in overall diversity.

## 2. Materials and Methods

### 2.1. Test Samples

In July 2022, surface soil samples were collected from five representative regions in the desert areas of the Tarim Basin: eastern (Shaya County), western (Qiemo County), southern (Yutian County), northern (Aral City), and central (Luopu County) (Table 1). At each sampling site, three spatially independent subsamples (0–10 cm depth) were randomly collected within a 20 m × 20 m plot. These subsamples were combined into a single composite sample per site to represent local heterogeneity. The resulting five composite samples (A–E) were treated as independent biological replicates and were processed separately throughout all subsequent cultivation and sequencing analyses, without mixing between sites.

Each composite sample was generated by combining three subsamples collected within a site and was treated as an independent biological replicate.

### 2.2. Cultivation and Enrichment Design

To evaluate how different levels of exogenous nutrient supply influence cultivation-driven restructuring of desert soil bacterial communities, this study established an amplicon sequencing dataset including one original soil sample group (Soil) and three enrichment-derived sample groups generated under different cultivation treatments. The original soil sample was used as the uncultured baseline reference. DNA was directly extracted prior to cultivation to represent the initial bacterial community structure.

The enrichment experiment included three cultivation treatments: standard R2A medium (R2A), diluted 100 times R2A medium (DR2A), and sterile water microcosms (Water). The sterile water microcosms consisted of laboratory-grade deionized water, which was sterilized by autoclaving (121 °C, 20 min) prior to use. No environmental water samples were used. Among the three treatments, R2A represented the baseline low-nutrient condition, DR2A represented a reduced nutrient supply level, and Water represented an extreme oligotrophic condition. Because R2A, although commonly considered a low-nutrient medium, still provides externally supplied nutrients, it was treated here as a relative baseline within a gradient of decreasing exogenous nutrient supply rather than as a strict simulation of in situ desert oligotrophic conditions. Together with diluted R2A (DR2A) and sterile water microcosms, it forms a gradient of decreasing nutrient supply.

For enrichment cultivation, soil inoculum was introduced into each of the three cultivation systems and incubated at 30 °C with shaking at 120 rpm. After incubation, microbial biomass from each treatment was collected for DNA extraction and 16S rRNA gene amplicon sequencing. Therefore, bacterial community analyses in this study were conducted across four analytical sample groups: Soil, R2A, DR2A, and Water. Among these, R2A, DR2A, and Water represent cultivation treatments, whereas Soil serves as the uncultured baseline reference and is not a cultivation treatment.

By comparing the original soil samples with enrichment cultures generated under different oligotrophic supply conditions, and further analyzing variations in main analyses were conducted at the treatment-group level, whereas inoculum size was treated as a secondary design factor and is not emphasized in the main results, this study aims not only to evaluate the impact of oligotrophic supply intensity on cultivation-driven selection effects, but also to disentangle the relative contributions of cultivation itself and nutrient supply intensity, and to determine whether graded oligotrophic conditions impose distinct and non-linear constraints on microbial community restructuring.

### 2.3. DNA Extraction and 16S rRNA Gene Amplicon Sequencing

Total genomic DNA was extracted from 0.5 g of soil or enrichment samples using the MP Soil DNA Kit (MP Biomedicals, Santa Ana, CA, USA) according to the manufacturer’s protocol. DNA concentration and purity were assessed using a NanoDrop 2000 spectrophotometer (Thermo Scientific, Waltham, MA, USA) and further verified by 1.2% agarose gel electrophoresis. The bacterial 16S rRNA gene was amplified using primer pair 799F (5′-ACTCCTACGGGAGGCAGCA-3′) and 1193R (5′-GGACTACHVGGGTWTCTAAT-3′), targeting the V5–V6 region. PCR amplification was performed in 25 μL reaction mixtures containing 12.5 μL of 2× PCR Master Mix, 1 μL of each primer (10 μM), 2 μL of template DNA, and nuclease-free water to the final volume. The thermal cycling program consisted of an initial denaturation at 98 °C for 2 min, followed by 25 cycles of denaturation at 98 °C for 15 s, annealing at 55 °C for 30 s, and extension at 72 °C for 30 s, with a final extension at 72 °C for 5 min. Amplicon libraries were prepared using sample-specific barcode/index sequences and Illumina adapter sequences, followed by paired-end sequencing on an Illumina MiSeq platform (Illumina, San Diego, CA, USA) with 2 × 300 bp chemistry.

### 2.4. Bioinformatic Processing

Raw sequencing reads were demultiplexed and processed using QIIME2 (version 2023.2) [19]. Primer trimming and quality filtering were performed prior to denoising using the DADA2 algorithm implemented in QIIME2 to infer amplicon sequence variants (ASVs). Chimeric sequences were identified and removed during denoising.

Taxonomic classification was performed using a naïve Bayesian classifier trained against the SILVA database (release 138.2) [20]. Phylum names follow the nomenclature of the SILVA database (release 138.2), while taxonomic names follow the SILVA 138.2 framework throughout the manuscript. Non-bacterial sequences (e.g., chloroplast and mitochondrial reads) were removed.

ASV tables were rarefied to the minimum sequencing depth across samples prior to diversity analyses.

All downstream analyses, including diversity metrics and network construction, were conducted at the ASV level.

### 2.5. Microbial Co-Occurrence Network Analysis

To explore potential co-occurrence relationships among microbial taxa under different cultivation conditions, correlation-adjusted microbial co-occurrence networks were constructed based on ASV data derived from 16S rRNA gene amplicon sequencing. Prior to network construction, the ASV abundance table was pre-filtered to retain only those ASVs with a relative abundance greater than 0.001 in at least one sample, thereby minimizing the impact of low-abundance or sporadic taxa on spurious correlations. The filtered ASV table was transposed and used for subsequent correlation analysis [21,22]. Pairwise correlations among ASVs were calculated using Spearman’s rank correlation coefficient, with parallel computing applied to improve computational efficiency. For each ASV pair, both the correlation coefficient (ρ) and its corresponding *p*-value were calculated. The Benjamini–Hochberg method was used to adjust *p*-values for multiple comparisons [23].

In the network construction, only the correlations that satisfy |ρ| ≥ 0.6 and have a corrected *p*-value < 0.01 are retained, and the rest of the correlations are excluded. Based on the filtered correlation matrix, an undirected weighted network is constructed, and the network is generated using the igraph package in the R language. During the construction process, self-related edges are removed, and isolated nodes with a degree of 0 are deleted. In the co-occurrence network, nodes represent bacterial taxa (ASVs), and edges represent statistically significant correlations between them. Edge weights correspond to the absolute values of the correlation coefficients, with the sign of the correlation (positive or negative) retained as an edge attribute. Taxonomic annotations at the phylum, class, order, family, genus, and species levels were mapped to each network node based on classification results. The resulting node and edge attribute tables were used for downstream network visualization and comparative analyses. It should be noted that correlation-based co-occurrence networks reflect statistical associations among taxa and do not imply direct ecological interactions or causal relationships.

### 2.6. Statistical Analysis

All statistical analyses were performed in R (version 4.3.0). Alpha-diversity indices (ACE and Shannon) were compared among treatments using the Kruskal–Wallis test. When significant, pairwise differences were assessed using Dunn’s post hoc test with *p*-values adjusted for multiple comparisons (Benjamini–Hochberg method). A significance threshold of *p* < 0.05 was applied.

Data are presented as boxplots, where boxes represent the interquartile range (IQR), the center line indicates the median, and whiskers denote 1.5 × IQR. Different letters above boxes indicate statistically significant differences among treatments (*p* < 0.05), whereas “ns” denotes non-significant differences.

## 3. Results

### 3.1. Bacterial Community Structure

#### 3.1.1. Phylum-Level Composition

**Figure 1:**
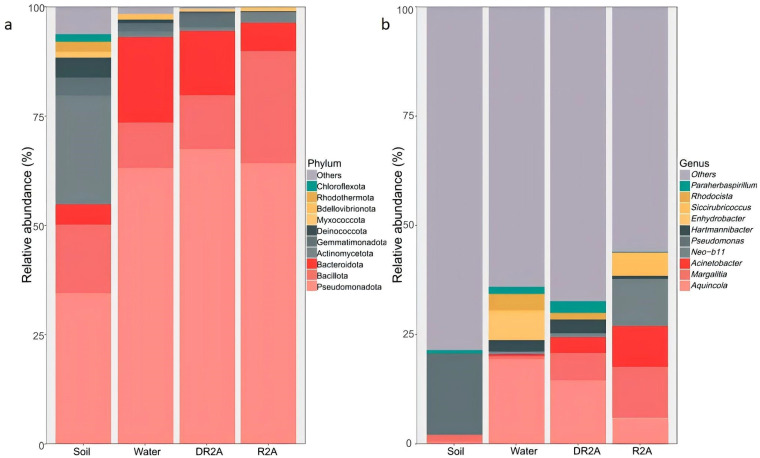
Relative abundance of bacterial communities across treatments at the phylum (**a**) and genus (**b**) levels under an oligotrophic nutrient gradient. Treatments include Soil, Water, DR2A, and R2A. Soil represents the original, uncultured samples, whereas R2A, DR2A, and water represent cultivation treatments. Only dominant taxa (relative abundance ≥ 1% in at least one sample) are shown individually, while low-abundance taxa (relative abundance < 1%) are grouped as “Others”.

Soil samples represent the original uncultured communities, while R2A, DR2A, and water correspond to enrichment-derived communities under different nutrient supply conditions. To evaluate how an oligotrophic nutrient gradient shaped bacterial community composition, we compared phylum-level profiles across treatments representing decreasing nutrient availability (R2A → DR2A → Water; Figure 1a). Across all treatments, Pseudomonadota was the dominant phylum, and its relative abundance increased along the enrichment gradient compared with the original soil. This increase was most pronounced in the R2A treatment, indicating that even under oligotrophic culture conditions, certain taxa were strongly favored during cultivation. Bacillota and Bacteroidota were the next most abundant phyla and exhibited differential responses along the nutrient gradient. Compared with R2A, both phyla contributed relatively more to the community in DR2A and Water, suggesting that reduced nutrient availability allowed a broader set of taxa to persist during enrichment.

In contrast, the original soil samples showed a more even distribution of phyla, with a higher representation of low-abundance groups, including Actinomycetota, Gemmatiomonadota, Deinococcota, Myxococcota, and Bdellovibrionota. These groups were substantially reduced across all enrichment treatments, regardless of nutrient level, indicating a strong cultivation-associated filtering effect.

Overall, phylum-level composition suggests that cultivation imposed a primary selection pressure, while decreasing nutrient availability within the oligotrophic gradient modulated the relative contributions of dominant phyla rather than fundamentally altering community structure.

#### 3.1.2. Genus-Level Composition

At the genus level, compositional differences among treatments became more pronounced (Figure 1b). The Soil samples were characterized by a high proportion of the Others category, representing numerous low-abundance genera rather than unclassified taxa. In this study, dominant taxa were defined as those with a relative abundance ≥ 1% in at least one sample, whereas low-abundance taxa were defined as those with a relative abundance < 1%. This pattern indicates a highly diverse community lacking strong dominance by a few taxa. In contrast, enrichment treatments exhibited a marked reduction in the proportion of Others, together with the emergence of a limited number of dominant genera.

Across the enrichment treatments, *Aquincola*, *Margalitia*, and *Acinetobacter* were among the most abundant genera, but their relative contributions varied along the oligotrophic nutrient gradient. *Aquincola* showed a marked increase in all enrichment groups compared with the soil samples and was particularly abundant in the Water and DR2A treatments, indicating that this genus responded strongly under more nutrient-limited conditions. *Margalitia* was also consistently enriched after cultivation and remained one of the dominant genera across all enrichment treatments. In contrast, *Acinetobacter* reached its highest relative abundance in the R2A treatment, suggesting that relatively higher nutrient availability within the enrichment gradient favored its proliferation.

Several other genera, including *Pseudomonas*, *Enhydrobacter*, *Rhodocista*, *Hartmannibacter*, *Siccibubricoccus*, *Pararhizobium*, and *Neo-b11*, were also detected across treatments, although their relative abundances were generally lower than those of the dominant genera. Compared with the original soil, the cultivated communities exhibited a more simplified genus-level structure, with fewer dominant genera accounting for a larger proportion of the total sequences.

These results indicate that the oligotrophic nutrient gradient exerted stronger selective effects at the genus level than at the phylum level. Cultivation broadly reduced genus-level complexity, whereas variation in nutrient availability further influenced the relative dominance of specific enriched genera.

#### 3.1.3. Differential Taxa Analysis

**Figure 2:**
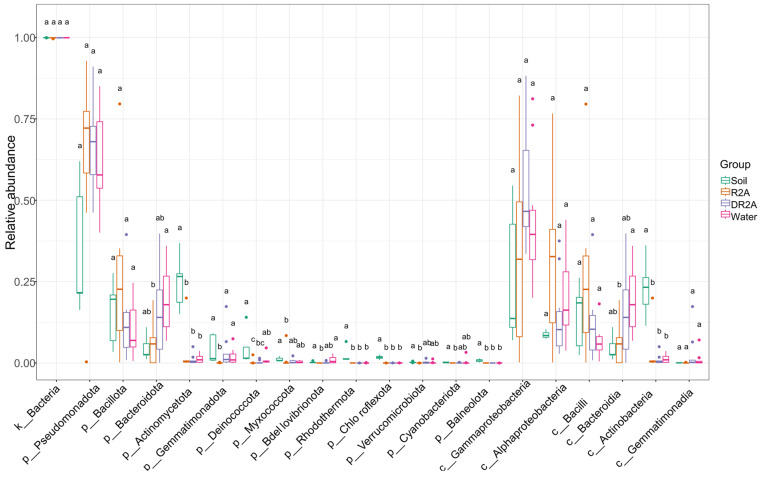
Differential abundance of dominant bacterial taxa across treatments at multiple taxonomic levels. The x-axis shows selected taxa at the kingdom (k__), phylum (p__) and class (c__) levels, allowing comparison of community shifts across taxonomic ranks. The y-axis represents relative abundance (%). Boxplots illustrate variation across five biological replicates (sampling sites) for each treatment, including soil (original samples), R2A, oligo (diluted R2A), and water (sterile water microcosms). Soil represents the original, uncultured samples, whereas R2A, DR2A, and water represent cultivation treatments. Different letters (e.g., a, b, c) above the boxes indicate statistically significant differences among treatments (*p* < 0.05), based on the Kruskal–Wallis test followed by Dunn’s post hoc test with Benjamini–Hochberg correction. Treatments sharing at least one letter are not significantly different, whereas treatments with no letters in common differ significantly.

Differential abundance analysis revealed treatment-dependent variation in microbial taxa across multiple taxonomic levels (Figure 2). To provide a comprehensive overview, dominant taxa from the kingdom, phylum, and class levels were presented together.

At the kingdom level, the relative abundance of k__Bacteria remained consistently high across all treatments and did not differ significantly, reflecting the overall stability of total bacterial abundance. At the phylum level, Pseudomonadota was the dominant group across all treatments and showed no significant differences, indicating a stable contribution to community composition. In contrast, Bacillota exhibited moderate variation, with relatively higher abundance in enrichment treatments compared to the original soil. Bacteroidota also displayed variability among treatments, with enrichment conditions generally showing increased abundance relative to soil samples. Several low-abundance phyla, including Gemmatiomonadota, Deinococcota, and Myxococcota, showed reduced relative abundances in enrichment treatments, suggesting sensitivity to cultivation conditions and potential loss during enrichment. At the class level, Gammaproteobacteria remained consistently dominant and did not differ significantly among treatments, mirroring the stability observed at the phylum level. In contrast, Alphaproteobacteria, Bacteroidia, and Bacilli exhibited greater variability across treatments. Notably, Bacilli showed relatively higher abundance under R2A conditions, indicating a response to comparatively higher nutrient availability within the oligotrophic gradient.

Overall, while dominant taxa remained relatively stable across treatments, several subordinate taxa exhibited significant but moderate shifts in response to cultivation and nutrient conditions, reflecting selective enrichment effects along the nutrient supply gradient.

### 3.2. Alpha- and Beta-Diversity

**Figure 3:**
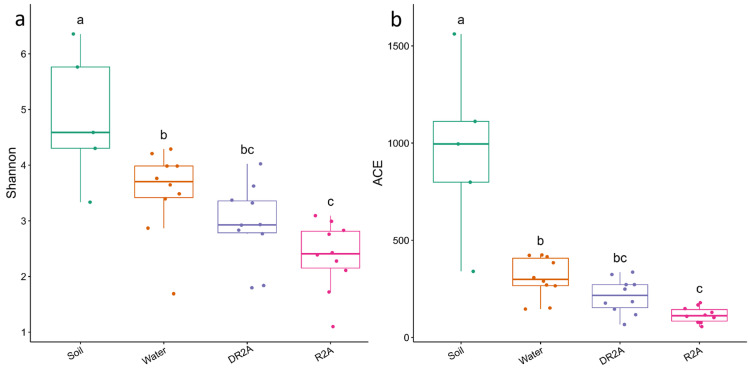
Alpha diversity indices of microbial communities across treatments. (**a**) Shannon diversity and (**b**) ACE richness. Different letters indicate significant differences among treatments (*p* < 0.05, Kruskal–Wallis with Dunn’s post hoc test).

Alpha diversity analysis revealed differences in bacterial richness and diversity among sample groups (Figure 3a,b). The Shannon index showed that the original soil samples generally maintained higher diversity than the enrichment-derived communities. Among the enrichment groups, Water tended to retain higher Shannon diversity than DR2A and R2A, whereas R2A generally showed the lowest values. The ACE richness index showed a similar pattern, with Soil exhibiting the highest richness overall, followed by Water, while DR2A and R2A displayed comparatively lower richness. These patterns suggest that enrichment cultivation reduced both the richness and diversity of bacterial communities relative to the original soil, and that this reduction tended to be stronger under higher exogenous nutrient supply.

**Figure 4:**
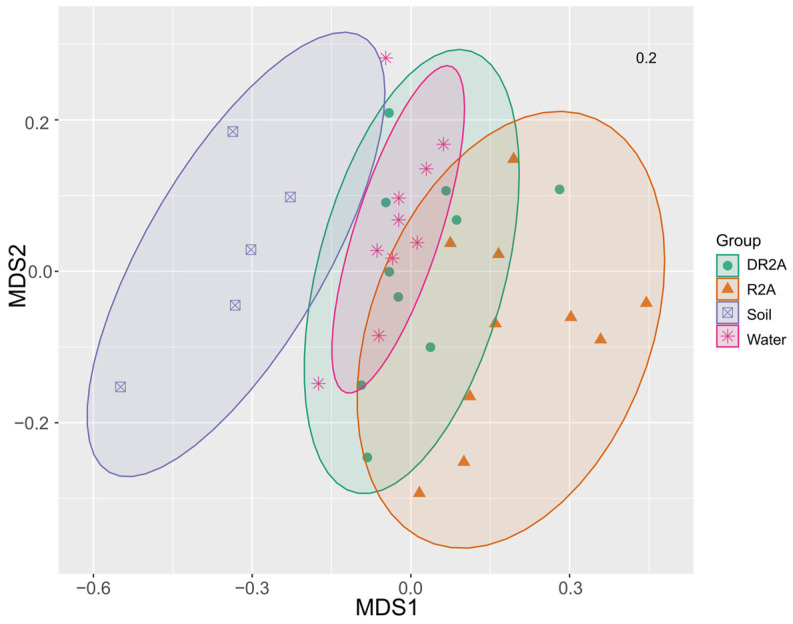
Non-metric multidimensional scaling (NMDS) ordination based on Bray–Curtis dissimilarity showing differences in bacterial community composition among treatments. Each point represents a sample, and ellipses indicate 95% confidence intervals. The stress value (0.20) indicates an acceptable representation of the data.

Beta diversity was assessed using Bray–Curtis dissimilarity and visualized by non-metric multidimensional scaling (NMDS) ordination (Figure 4). The ordination showed that the original soil samples were positioned separately from the enrichment-derived communities, indicating an overall cultivation-associated shift in community composition. In contrast, the Water, DR2A, and R2A groups exhibited substantial overlap, with only limited separation in ordination space, suggesting that differences in nutrient supply intensity had a relatively modest effect on overall community structure. Collectively, these results indicate that cultivation was the dominant factor associated with community reassembly, whereas nutrient supply intensity acted as a secondary factor influencing enrichment-derived community variation.

### 3.3. Network Topology and Topological Roles Across Nutrient Supply Levels

Co-occurrence network analysis was performed to evaluate microbial interaction patterns under different nutrient supply levels (Figure S1). The overall network structure differed markedly between the original soil and enrichment treatments, with the soil network exhibiting substantially higher complexity than all enrichment-derived networks. This pattern was quantitatively supported by network topological properties (Table S1). The soil network contained the largest number of nodes (1158) and edges (69,829), along with the highest average degree (120.60) and network density (0.1042), indicating a highly connected and complex interaction network. In contrast, all enrichment-derived networks showed pronounced reductions in node number, edge number, and connectivity, reflecting a substantial simplification of microbial interaction structure following cultivation.

**Figure 5:**
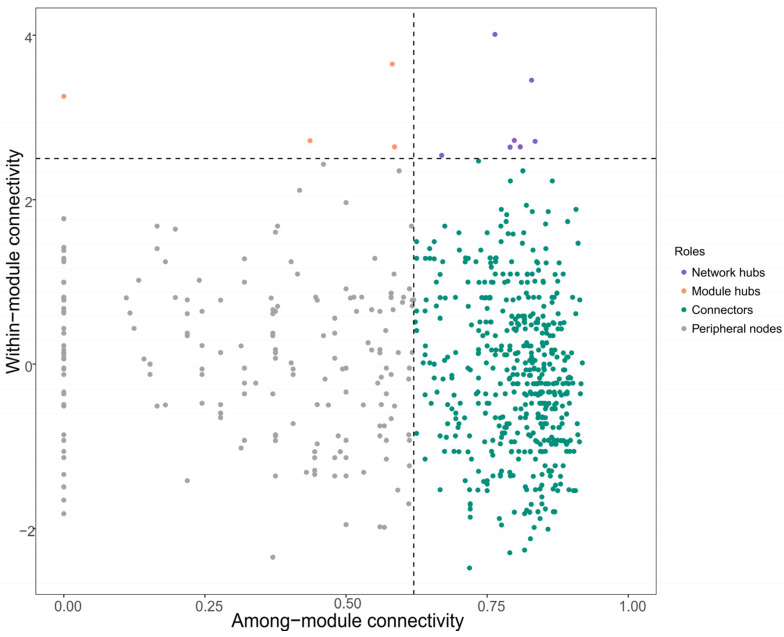
Topological roles of microbial taxa inferred from co-occurrence networks. Nodes are classified based on within-module connectivity (Zi) and among-module connectivity (Pi). Thresholds of Zi = 2.5 and Pi = 0.62 were used to define peripheral nodes, connectors, module hubs, and network hubs. Each point represents an individual taxon.

Among the enrichment treatments, differences in network topology were comparatively modest. DR2A and Water exhibited slightly higher modularity (0.8508 and 0.8409, respectively) than R2A (0.7739), suggesting a tendency toward more compartmentalized network organization under lower nutrient supply. However, other metrics, including average degree and density, showed only limited variation across enrichment treatments, indicating that nutrient supply intensity had a relatively minor effect on overall network complexity compared with the strong filtering imposed by cultivation. To further assess the functional roles of taxa within these networks, Zi–Pi analysis was conducted (Figure 5). Across all treatments, the majority of nodes were classified as peripheral nodes, with only a small proportion identified as connectors, and no module hubs or network hubs were detected. This distribution indicates that most taxa were weakly connected within modules and had limited influence on global network connectivity. Importantly, the Zi–Pi patterns were highly consistent across nutrient treatments, further supporting the conclusion that variation in nutrient supply intensity had only a limited effect on higher-order interaction patterns among taxa.

Taken together, the structural (Figure S1), quantitative (Table S1), and topological role (Figure 5) analyses consistently indicate that cultivation substantially reduces microbial network complexity and connectivity, whereas nutrient supply acts only as a secondary modulator, with modest effects on network organization and taxon-level roles. Network construction parameters were standardized across treatments to allow relative comparison of topological properties.

## 4. Discussion

### 4.1. Cultivation Imposes a Dominant Deterministic Filter on Community Assembly

The present study demonstrates that cultivation acts as a dominant deterministic filter, driving pronounced restructuring of soil microbial communities across all treatments.

Across taxonomic levels, enrichment resulted in a substantial reduction in community complexity, characterized by the loss of numerous low-abundance taxa and the emergence of a limited number of dominant lineages. This pattern is consistent with previous studies showing that only a small fraction of environmental microorganisms can be recovered through cultivation, with strong selection for fast-growing and metabolically versatile taxa [13].

The clear separation between soil and enrichment-derived communities in NMDS space further supports this interpretation, indicating that community reassembly is primarily driven by cultivation-associated environmental shifts rather than intrinsic soil heterogeneity. Such shifts likely reflect the removal of spatial structure and microscale environmental gradients that are critical for maintaining microbial diversity in natural soils [24,25].

The dominance of Pseudomonadota across treatments is also consistent with prior enrichment studies, where members of this phylum frequently proliferate because of their metabolic flexibility and rapid growth under conditions [15,26].

Together, these results support a deterministic assembly framework, in which cultivation imposes a strong environmental filter that overrides stochastic processes and constrains community composition [17,27].

### 4.2. Nutrient Supply Intensity Acts as a Secondary Ecological Filter

Within the overarching cultivation filter, the oligotrophic nutrient gradient (R2A → DR2A → Water) exerted a secondary but detectable influence on community composition.

At the phylum level, relatively limited variation was observed across treatments, suggesting that broad taxonomic structure is robust to moderate variation in nutrient supply. In contrast, genus-level composition showed clearer differentiation, indicating that nutrient availability primarily influences fine-scale ecological niches rather than higher-level taxonomic organization [28].

The differential responses of enriched genera along the nutrient gradient likely reflect resource-based niche partitioning, where taxa differ in their ability to exploit limited versus relatively enriched substrates [29,30]. Importantly, these shifts occurred within a shared pool of enriched taxa, indicating that nutrient supply modulates competitive hierarchies rather than determining initial taxonomic selection.

This pattern is consistent with ecological theory predicting that environmental filters operate hierarchically, with stronger filters determining community membership and weaker filters influencing relative abundance [31].

### 4.3. Diversity Loss Reflects Selective Exclusion and Competitive Dominance

The reduction in alpha diversity following enrichment highlights the strong selective pressure imposed by cultivation.

Both Shannon diversity and ACE richness declined markedly from soil to enrichment treatments, indicating losses in both taxonomic richness and evenness. Such reductions are widely reported in enrichment systems and are typically attributed to competitive exclusion under simplified and resource-defined environments [32,33].

Interestingly, diversity tended to be higher under Water conditions compared with DR2A and R2A, suggesting that lower nutrient availability may reduce competitive asymmetry, thereby allowing coexistence of a broader range of taxa. This aligns with theoretical and empirical evidence that resource limitation can promote diversity by weakening dominance effects [34,35].

However, the relatively modest differences among enrichment treatments indicate that nutrient-driven diversity effects are constrained by the stronger influence of cultivation, reinforcing the hierarchical filtering framework.

### 4.4. Weak Differentiation in Beta Diversity Indicates Constrained Community Space

Despite observable taxonomic shifts, beta diversity analysis revealed substantial overlap among enrichment treatments, indicating limited differentiation in overall community structure.

This suggests that community assembly is constrained within a reduced compositional space defined by cultivation, where different nutrient conditions primarily influence relative abundance rather than generating distinct community states. Similar patterns have been observed in simplified microbial systems, where strong environmental filtering limits community divergence [15,26].

Thus, while nutrient supply contributes to compositional variation, it does not fundamentally alter the trajectory of community assembly under cultivation conditions.

### 4.5. Simplified Co-Occurrence Networks Reflect Loss of Ecological Complexity

Co-occurrence network analysis revealed that microbial communities across all treatments were characterized by simplified topological structures dominated by peripheral nodes, with few connectors and no detectable hubs.

In natural soils, microbial networks often exhibit complex architectures with highly connected hub taxa that contribute to stability and functional integration [36,37]. The absence of such structures in this study suggests that cultivation disrupts complex ecological interactions, likely through the loss of rare taxa and the breakdown of spatially structured niches.

Moreover, the similarity of network topology across nutrient treatments indicates that nutrient supply intensity has limited influence on higher-order interaction patterns, further supporting the conclusion that cultivation is the primary driver shaping community organization.

### 4.6. A Hierarchical Framework for Microbial Assembly Under Oligotrophic Cultivation

Integrating taxonomic, diversity, and network analyses, our results support a hierarchical model of microbial community assembly under oligotrophic cultivation conditions:

Primary filter: Cultivation environment;

Secondary filter: Nutrient supply gradient;

Outcome: Simplified communities with reduced interaction complexity.

This framework is consistent with contemporary ecological theory that emphasizes the importance of multi-level environmental filtering in shaping microbial communities [17,38].

### 4.7. Implications for Interpreting Enrichment-Based Microbial Studies

These findings highlight several important implications. Enrichment-derived communities cannot be directly considered proxies for natural systems, given the strong cultivation bias that reshapes microbial composition [13]. Within this constrained framework, nutrient gradients act primarily as secondary modulators of community assembly, rather than as primary determinants, emphasizing the hierarchical nature of ecological filtering. Moreover, the simplified network structures observed here suggest that interaction patterns inferred from enrichment systems may underestimate the complexity and connectivity characteristic of natural microbial communities [36,37].

## 5. Conclusions

In this study, we evaluated how an oligotrophic nutrient gradient influences cultivation outcomes and enrichment-derived bacterial community structure in desert soils from the Taklimakan Desert. By integrating cultivation-based enrichment with amplicon sequencing and network analysis, we showed that cultivation itself was the dominant selective force shaping microbial community composition, whereas nutrient supply intensity acted as a secondary modulator of enrichment outcomes.

Compared with the original soil communities, all enrichment treatments exhibited clear compositional restructuring and reduced alpha diversity, indicating strong cultivation-associated filtering. Within the enrichment system, variation in nutrient supply further influenced the relative abundance of specific taxa, with treatment-dependent responses being more evident at the genus level than at higher taxonomic levels. In contrast, overall beta diversity patterns and network topology differed only modestly among the enrichment treatments, suggesting that nutrient supply had a limited effect on higher-order community organization.

Overall, our findings indicate that nutrient supply should not be regarded merely as a technical detail in cultivation design, but as an ecologically relevant factor that modulates which taxa are preferentially enriched under conditions. At the same time, the results emphasize that cultivation bias remains the primary driver of divergence between enrichment-derived and natural soil communities. Incorporating nutrient gradients into cultivation systems may therefore improve the ecological interpretation of enrichment outcomes and provide a useful framework for refining cultivation strategies for oligotrophy-associated microorganisms in resource-limited desert ecosystems.

## Figures and Tables

**Table 1:** Sampling sites and composite sample structure.

Site ID	Location	Coordinates (E, N)	Altitude (m)	Composite Sample
A	Yutian County	81°17′40″ E, 36°54′09″ N	1364.0	Soil_A
B	Alar City	82°05′59″ E, 40°48′00″ N	926.0	Soil_B
C	Qiemo County	83°48′00″ E, 39°18′50″ N	990.0	Soil_C
D	Shaya County	82°19′41″ E, 39°37′52″ N	988.0	Soil_D
E	Lop County	80°57′25″ E, 39°4′59″ N	1128.5	Soil_E

## Data Availability

The datasets generated for this study are available in the NCBI Sequence Read Archive (SRA) under BioProject accession number PRJNA1405623.

## Supplementary Materials

The following supporting information can be downloaded at https://www.mdpi.com/article/10.3390/biology15100755/s1: Figure S1: Co-occurrence network structures under different treatments: (a) Soil; (b) Water; (c) DR2A; (d) R2A; Table S1: Topological properties of co-occurrence networks under different nutrient supply treatments.
