# Supplementary material for: Nutrient Supply Gradients Modulate Cultivation-Driven Restructuring of Microbial Communities in Desert Soils
Source: Biology (Basel). 2026 May 9;15(10):755. doi: 10.3390/biology15100755 (PMC13203477; doi:10.3390/biology15100755)
Supplement: Supplementary file 1 [file biology-15-00755-s001.zip › biology-4292759-supplementary.pdf]

biology MDPI

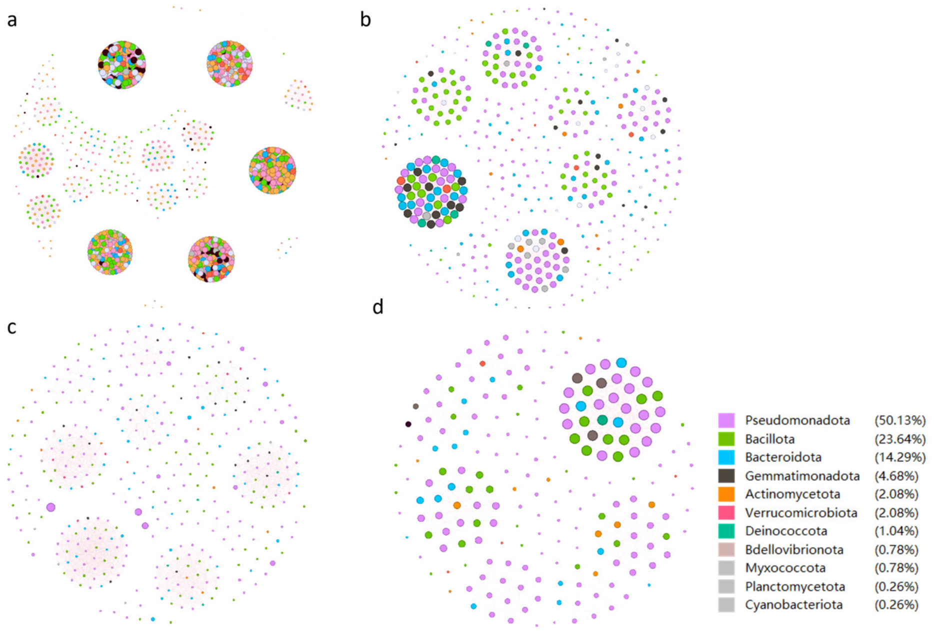

Figure S1. Co-occurrence network structures under different treatments:(a) Soil; (b) Water; (c) DR2A; (d) R2A.

Table S1. Topological properties of co-occurrence networks under different nutrient supply treatments.

Parameter	Soil	Water	DR2A	R2A
Number of nodes	1158	455	388	205
Number of edges	69829	4210	4116	1447
Average degree	120.6	18.51	21.22	14.12
Network density	0.1042	0.0408	0.0548	0.0692
Average path length	1	1.1	1.01	1
Network diameter	1	6	4	2
Clustering coefficient	1	0.9994	0.9998	0.9999
Modularity	0.8058	0.8409	0.8508	0.7739
Heterogeneity	0.5741	0.8893	0.7328	0.7954
Centralization	0.0626	0.0716	0.064	0.0975

Topological properties of co-occurrence networks corresponding to Figure S1.

Disclaimer/Publisher's Note: The statements, opinions and data contained in all publications are solely those of the individual author(s) and contributor(s) and not of MDPI and/or the editor(s). MDPI and/or the editor(s) disclaim responsibility for any injury to people or property resulting from any ideas, methods, instructions or products referred to in the content.
